# Learning from success stories when using eLearning and bLearning modalities in higher education: a meta-analysis and lessons towards digital educational transformation

**DOI:** 10.1186/s41239-022-00325-x

**Published:** 2022-04-12

**Authors:** Álvaro H. Galvis, Diógenes Carvajal

**Affiliations:** 1 Conecta-TE, Universidad de los Andes, Bogotá, Colombia; 2 Centro de Innovación en Tecnología y Educación, Bogotá, Colombia; 3 Centro de Ensañanza Aprendizaje y Trayectoria Profesional, Colegio Mayor de Nuestra Señora del Rosario, Bogotá, Colombia

**Keywords:** bLearning (blended learning), eLearning (electronic learning), Educational modalities, Case-based meta-analysis, Educational transformation with technology, Key success factors, Educational change management, Educational innovation

## Abstract

This work seeks to support scholars interested in non-face-to-face modalities of higher education in making decisions about the use of digital and educational technologies (DET) to promote educational transformation (ET) in the context of their organizations. This organizational change deals with the implementation of technology-based flexible educational practices, focused on helping students develop competencies of interest for their personal and professional growth. With this in mind, in 2018 we identified and followed six leading higher education institutions on three continents that, for years, have carried out educational innovation experiences with the support of technology. Two of the analyzed experiences make use of eLearning, another of bLearning, and the others combine eLearning and bLearning as a complement to the face-to-face modality. The meta-analysis of the cases, carried out in 2019, followed suggestions from (Stake in The art of case study research, Sage Publications Inc., 1995) as well as from qualitative research that seeks to understand what is behind the cases from three dimensions: education, technology, organization. For each one, we determined what they do, how they do it, and what success factors must be considered. As the data was collected before the 2020 pandemic and this issue produced structural imbalances in society and in higher education, it was considered pertinent, at the end of 2020, to check the pulse of the ET mediated with DET in three of the six institutions studied. The purpose was to refine the findings of the meta-analysis and learn from the decisions made in the situation of forced change in environments, means, and strategies to continue providing quality higher education.

## Introduction

This work fills a gap in the DET-mediated change management literature that aims to produce ETs in higher education (Galvis, 2018). It occurs at a time of inflection in the curve of institutional changes, since the improvement dynamics that each higher education institution (HEI) brought pre-pandemic was affected by the need to provide remote education and, progressively, move towards a “new normal” (Lopera, 2020). It builds knowledge on educational, technological and organizational findings of the HEIs whose cases were documented by Galvis (2019). It leaves very important lessons in the strategic, tactical and operational aspects of an ET mediated with DET. In the six cases analyzed, institutional strategic thinking guides the digital transformation of academic processes. The educational dimension is the heart of these changes and the technological and the organizational dimensions are aligned and at the service of the former. In each of these dimensions, the study details the value added by the underlying components and clarifies those few things that one must take care of to be successful—the critical success factors. At the time of the pandemic, the importance of the level of institutional maturity towards the ET mediated with DET became evident (Marks et al., 2021), as well as the institutional enclave and strategy to achieve academic excellence (Lopera, 2020). Case studies are, by definition, particularities from which one can learn about what is being examined (Stake, 1995), in this study, good practices in digital transformation of higher education institutions; thus, the results of this study are not generalizable but illustrative. By reading them with a meta-case lens, an understanding of trends and tensions is gained, which helps to elucidate issues that it is good to take into account when trying to extrapolate findings to one's own context. The reader does not receive a recipe but rather principles and ideas that can guide his action, whilst taking into consideration the environment itself.

## Motivation and background for the study

The interest in carrying out a comparative study of success stories in the use of eLearning and bLearning in higher education institutions (HEIs) arises from the desire to support those who make decisions regarding how to be successful in the use of flexible learning environments supported by digital technologies (DT).

Having computers and the internet to support the substantive and operational work of HEIs is no longer an option. It is a strategic requirement to move forward in fulfilling the educational mission of organizations that, like many universities and advanced training centers, want to be in tune with the society they serve—the knowledge society. This should be done in the context of a global economy with a local enclave, where “human capital and information are paramount and in which the production system is organized based on information, knowledge, and technologies” (Galvis, 2018, p. 108).

The strategic use of DETs in higher education builds on the opportunities they provide to fulfill the mission of each HEI and by far exceeds the technological endowment, which is a necessary but not sufficient condition. What is involved with the strategic use of DET in HEI is for the institution to be successful in its mission. Within the principles and values that guide institutional action, DET should realize opportunities that allow value to be added to the beneficiaries, based on the role they play (Galvis, 2018). This is challenging, since each HEI is different from the others. This is due to its nature, to its socio-cultural, political, and economic framework, to the composition of its constituent groups, to the stakeholders that influence it, as well as because of the institutional strategies that guide the actions and with which it is expected that the use of DET will be aligned.

The rate of change in technological and educational products and processes is also challenging. Rate of change is increasingly rapid due to the pace of knowledge renewal; maintaining a rhythm of innovation that makes a difference in a sector such as higher education requires sharpness and agility in decision-making throughout the life cycle of the substantive processes, which in a HEI, we usually call academic and research.

The offer of advanced training opportunities goes far beyond HEIs, since there are also offerings from publishers, mass media, corporate universities, and many services for flexible and tailor-made education. On the other hand, given that resources are limited, it is necessary to take advantage of the educational opportunities offered by the DETs. Reasons such as these merit efforts like the meta-analysis presented in this study.

This meta-analysis builds on the findings of a study on good practices in the use of bLearning and eLearning in HEIs (Galvis, 2019). The HEIs studied include six cases, selected because of their exemplary use of one, or both, non-face-to-face educational modalities. Regardless their contextual differences (from six different countries in three continents) all of them deal with flexible learning environments in tertiary education, mediated with digital technologies. In addition, each of these cases belong to institutions with trajectory in technology-based educational change, meaning with this that technologies have been a means and student-centered pedagogy a common educational strategy.

1. The Open University of Catalonia—UOC. This was chosen as a case study for having the virtual modality inherent to its educational work and for having created and launched an educational model that serves as a reference for those who are interested in online higher education (Galvis & Duart, 2019).
2. The case of Babson College, headquartered in Massachusetts. BC case is very interesting because its Business Administration programs are very successful blended training experiences; they use the case method and are authentically evaluated (Galvis et al., 2019a).
3. The New Technologies Education Project (PENT acronym, in Spanish) of the Latin American Faculty of Social Sciences (FLACSO for its acronym, in Spanish), headquartered in Argentina. This is a very interesting case of eLearning as a successful experience of advanced training, which is carried out in cycles and focuses on authentic problems (Galvis et al., 2019c).
4. The Tecnológico de Monterrey—TEC—is an institution with face-to-face and virtual multicampus, with national and supra-national reach. There is a history of offering programs in the three educational modalities at Tecnológico de Monterrey. Its TEC21 Model is a very interesting example of ET (Valenzuela González & Galvis, 2019).
5. The Pontificia Universidad Católica del Perú (PUCP), a historically face-to-face university, with a century of experience and high recognition at the local and global level. This is a good case of ET with digital technologies. This case study focuses on the incorporation of eLearning and bLearning modalities (Galvis & Ugaz-Lock, 2019).
6. The case of Universidad de los Andes, in Bogotá. This is an interesting case study as an experience of maturation and institutionalization of transformative academic practices with the support of active pedagogies and DET. It is a university where the search for excellence and curricular flexibility have been a constant. The combination of virtual and blended modalities complements face-to-face teaching at Uniandes (Galvis et al., 2019c).

The literature and experience review allowed it to be highlighted that the possible hybridization, when combining educational modalities, when being transformative is the desire (Graham, 2006), must be multidimensional (Galvis, 2017; Singh, 2003). The blend should not be a simple mixture of face-to-face with virtual. This review also assisted to propose a methodology that, with a strategic approach, helps HEIs to assume the process of ET with DET as a process of cultural change. HEI reflect and take position on the educational approach, operational strategy, and economic models that determine different degrees and nuances of multidimensional hybridization to create and implement learning environments supported by DET (Galvis, 2019).

## Methodology

**Fig. 1:**
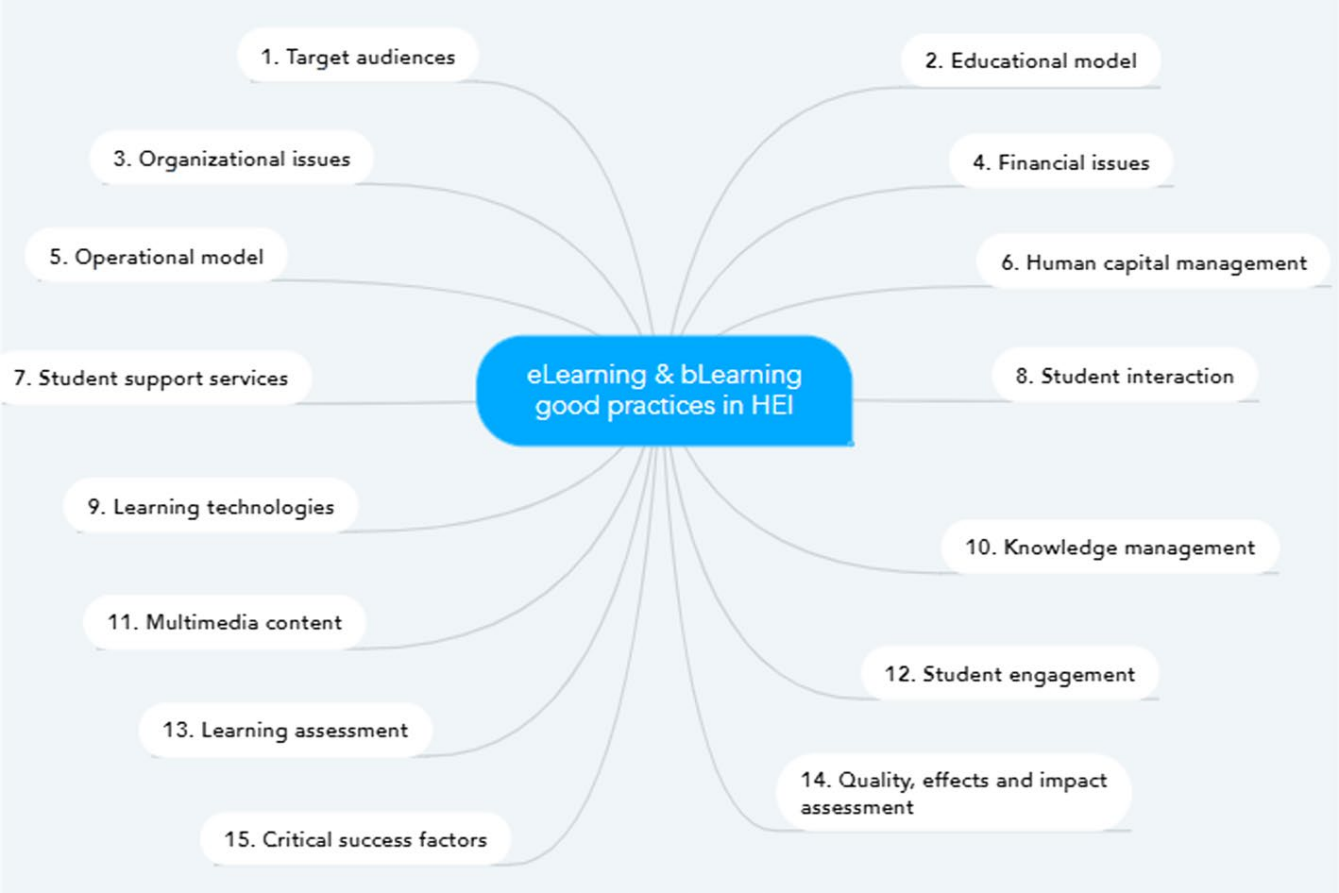
Mind map with objects of inquiry and analysis of good practices in eLearning and bLearning in HEI. Source: Translated from Galvis (2019, p. 116)

The aforementioned reasons are behind the selection of the six cases of institutions being studied, which cover five countries and three continents. A first version of the cases was proposed in 2012 from the analysis of digital information provided by key informants from each HEI invited to participate, as well as from structured interviews regarding variables relevant to the design and implementation of programs and courses in the virtual and hybrid modalities. The results of this study served as the basis for the findings analyzed in the benchmarking made by (Galvis & Pedraza, 2013). When it was decided to update this work in 2015, the literature review was renewed and Colombian experts in eLearning and bLearning were asked for feedback about the original interview guide. Fifteen objects of inquiry and analysis of good practices in eLearning and bLearning were established, as shown in the following mind map, which is explained in Galvis (2019) (Fig. 1).

In 2015, the original list of institutional contacts was enriched in order to update the six cases. In four of the studies, the information was updated from structured interviews [UOC, BABSON, PENT-FLACSO, PUCP], and in the other two, the case was built from scratch: one was authored by the key informant [Tecnológico de Monterrey], and the other from the interaction between those responsible for the case [UNIANDES], following the same structure of the other objects of study. Given the changes reported between the first and second iterations, in 2018 key informants were asked to review and update their case information. This information is shared in section 2 of Galvis (2019).

The meta-analysis of the six cases has been made following suggestions from (Stake, 1995), through qualitative research that seeks to understand what is behind the objects of study, from each of the dimensions that were examined. For this, a cross-sectional analysis of the six cases was carried out, in order to identify common elements and differentiating issues. For each of the cross-sectionally studied dimensions—educational, technological, organizational—we determined what they do, how they do it, and what success factors must be taken care of. The analysis did not imply the identification of consensus between the experiences, but it showed the understanding of the factors that guarantee an eLearning or bLearning program is successful, such as learning for other institutions.

Theme analysis was used to analyze the information gathered from each experience; the data were reviewed and categorized using ATLAS.ti software, and the information was fragmented into analysis units corresponding to ideas that were, then related among them using the principles of semantic analysis which resulted in mental structures.

The abrupt change to remote teaching that the COVID-19 pandemic in 2020 generated was a trigger for the acceleration of change and redirection, at times, to comply with the quality and safety conditions in higher education institutions (Lopera, 2020).

The findings, organized according to the aforementioned thematic structure, are presented below, and within each theme the key elements identified.

## Findings before the COVID-19 pandemia

This section presents mental structures resulting from reading the six cases documented before COVID-19 pandemia and studied transversely—as diagrams of relationships (verbalized on the arrows) between pairs of shared driving ideas (concepts in red) that are organized (concepts in white) or structured (in blue) ones. Throughout the writing, the educational, technological, and organizational findings are presented for each of the established core concepts, in the context of the digital transformation that each of the educational organizations under study has sought for years. Each of these main ideas is analyzed and exemplified.

### Findings concerning the educational dimension

Educational ideas prove to be the focus of the digital transformation in each of the experiences analyzed. What do they do in the educational dimension? How do they do it? What are they based on? What are the key success factors in this dimension?

#### What do these cases take care of from the educational perspective?

**Fig. 2:**
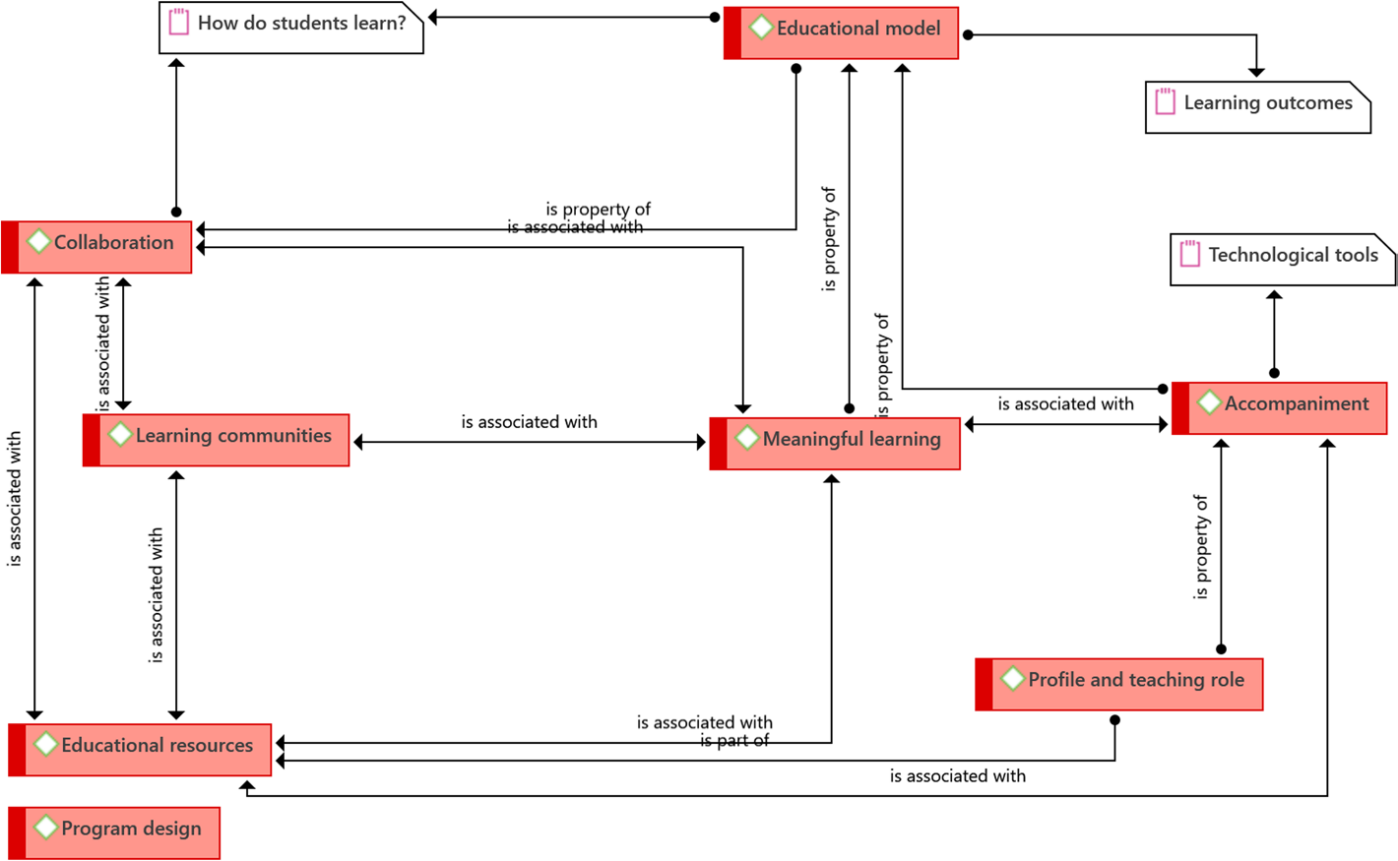
Structure of driving ideas that guide what they do in education. Source: Diógenes Carvajal

The first core idea that arises is the educational model, which is a central aspect for the design of the eLearning and bLearning programs. The existence of a learning model is necessary in which the key elements of virtual programs are defined. In the case of the UOC, for example, they refer to the confluence of learning resources, collaborative learning, and permanent support for students; in the case of the PUCP, the emphasis is on learning activities, student autonomy, and research and collaboration mediated by ICT. In this way, what they do from the educational perspective goes beyond the educational incorporation of technological tools and focuses on the definition of strategies focused on the teaching–learning processes (Fig. 2).

A second key element is Collaboration. When designing programs, the bet is on students learning collaboratively, and the technological tools facilitating such collaboration. It should be taken into account that collaboration cannot be made in isolation, but within the framework of an educational model and, likewise, inserted in a conception of how students learn. In the experiences studied, it was identified that there is a constructivist background that supports the proposition that people learn better in collaborative spaces in which activities are carried out that lead the student to build new knowledge upon old knowledge. This theoretical stance permeates the designs and tools used to achieve collaborative spaces in which students and their teachers share and discuss ideas, and there is permanent feedback. Collaborative work allows the creation of learning communities in which students will be grouped around a common learning objective, and will carry out tasks and activities that allow them to advance as a team in achieving them.

This leads to the identification of a third strong idea: meaningful learning. The idea of meaningful learning implies that students build new knowledge on previous knowledge (scaffolding), and that the knowledge built makes sense and is useful for the students. This aspect is shared by several of the institutions studied, stating the need to contextualize learning, and that real-life cases be studied to support the achievement of learning objectives. The center of the process is the development of learning activities. Creating spaces such as what PENT-FLACSO calls professional practice, whose objective is for students to go into the field and study real cases—which contributes to meaningful learning.

As mentioned above, collaboration also allows for the formation of Learning Communities, which is the fourth key element identified. In addition to the above, it should be noted that these communities are the result of the students' learning process, within the framework of their autonomy. They are supported by their teachers, classmates, and are assisted by the various technological tools available, as well as the spaces designed for the exchange of ideas. The formation of learning communities would not occur without the existence of all the other elements, within a pedagogical design that has the learning objectives that students are expected to achieve as its center.

A fifth element that emerges tangentially, but has an important impact, is accompaniment. During all planned activities, students have the permanent and close accompaniment of their teachers as guides who, as experts, support the learning process of students and are, likewise, part of the learning community that is being created and strengthened. Here, the technological tools that allow monitoring and feedback on the progress of students, both synchronous and asynchronous, and eventual face-to-face meetings in the case of programs that have it planned, add value. Successive reviews, dialogue and supervision by teachers or tutors are key so that the accompaniment contributes to the achievement of the learning objectives.

Similarly, the educational resources available will allow learning objectives to be achieved; these are the tools that enable meaningful learning, accompaniment, collaborative work, and the formation of learning communities. They cover not only the technological tools but also the activities, materials, people, and roles established within the design of the program. It could be argued that an educational resource is any activity or instrument designed that facilitates and supports the achievement of students’ learning objectives. This is the sixth element identified which, as seen, is a set of driving ideas that complement and interrelate among them.

Although the presence of the teacher or tutor has been mentioned as support for the students in their educational process, it was identified that it is necessary, in the design of the program, and within the framework of the pedagogical model, to define a profile and teaching role that guarantees the good progress of learning, which is the seventh element. Of the six cases studied, the Tecnológico de Monterrey study defines five attributes that teachers must have. They must be inspiring, at the forefront in their discipline, innovative, highly linked to the activities of their profession, and skillful in technology.

To finish this section, it should be taken into account that the elements presented so far converge in the program design. It could be said that the key to success lies in the design that is made from the beginning and in which the various tools, strategies, spaces, and roles have a place to achieve the learning objectives.

#### How do they do the educational component?

**Fig. 3:**
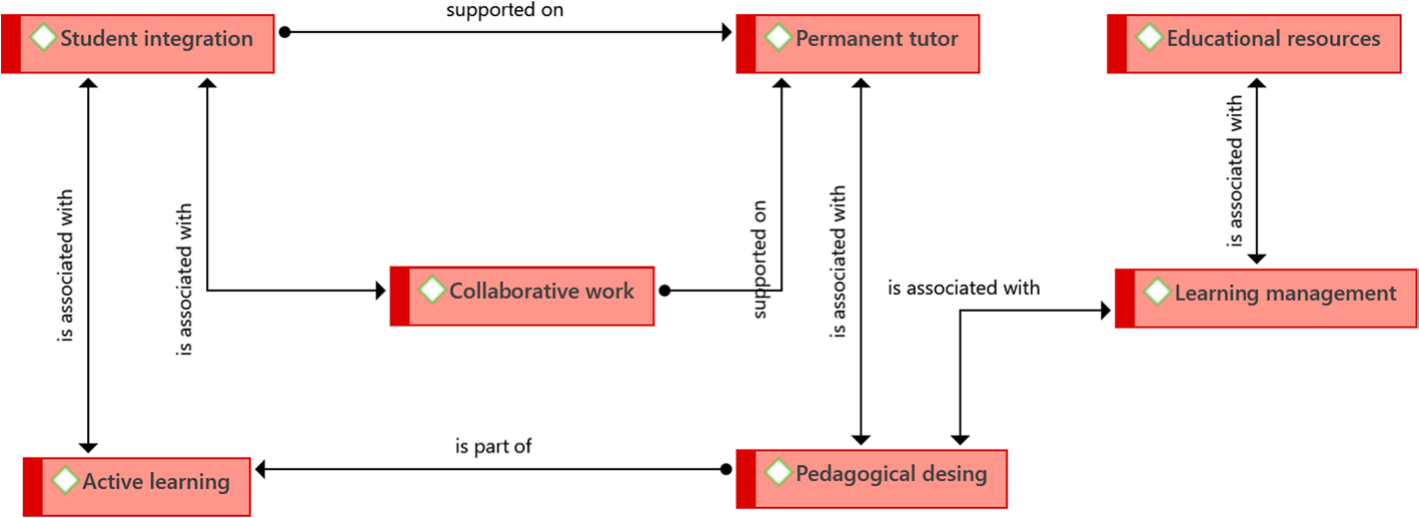
Structure of driving ideas that guide the educational component. Source: Diógenes Carvajal

Understanding how teach in these cases is structured around seven main ideas—some are related to those already identified in the previous section (Fig. 3).

The central core idea is collaborative work and the central role collaboration plays in the student learning process. It is a question of resorting to collaborative strategies, which, from the outset, have a particular view of the teaching–learning process, and are framed mainly in constructivist learning theories, with no exclusion of other possibilities (in the following section, it will be seen that the experience of Uniandes enables the convergence of active pedagogies with conventional ones).

Although it was identified in the previous section that a teacher profile and role must be defined, in this section it was found that it is convenient to have a permanent tutor, who can be the same teacher of a class, or another who does transversal monitoring. In the case of PENT-FLACSO, for example, the tutor remains throughout the learning process. Although the teachers who are responsible for the subjects may change, the tutor does not. So, he / she knows the permanent work of the student, not only in terms of their subject matter, but also in terms of their learning style and characteristics. The tutor provides personal accompaniment, and seeks to empower the student to generate autonomy in themselves and their own learning. The accompaniment includes the supervision of access to the learning environment, to the various tools and the performance of assigned tasks. Similarly, they communicate privately with the students (when they consider it necessary) and give them pertinent feedback. The accompaniment is transversal, permanent, and personalized, and, in a certain way, is shared by the students, to the extent that learning communities are formed in which their members take care of each other.

This last aspect relates to student integration, which consists of promoting communication between students and between them and the teacher in such a way that collaborative activities are favored. Specifically, Babson College, during its induction, aims to integrate blended learning students into the institution, just as it happens with face-to-face students. Student integration is configured as an element that affects the sense of belonging of students and their involvement in their own learning process, and it is part of the collaborative learning pedagogical strategy.

The fourth element identified was called learning management, and it expands the idea of educational resources, emphasizing the system that is implemented and designed (in technology) to house the tools that enable student learning as well as the pedagogical strategies that enable learning and interactions.

The fifth fundamental concept is active learning, closely related to meaningful learning. Institutions that propose active learning want students to apply their knowledge in a relevant and meaningful way outside the classroom, in their professional context, and there is a contextualization of learning. Thus, pedagogical strategies such as action research, case studies, and project-based learning are used.

A clear connection can be seen between the elements presented in this section and those corresponding to the previous section. The idea that all these elements are permanently present and interconnected throughout the learning process is reinforced by the idea of caring for educational design.

#### Key success factors in the educational dimension

**Fig. 4:**
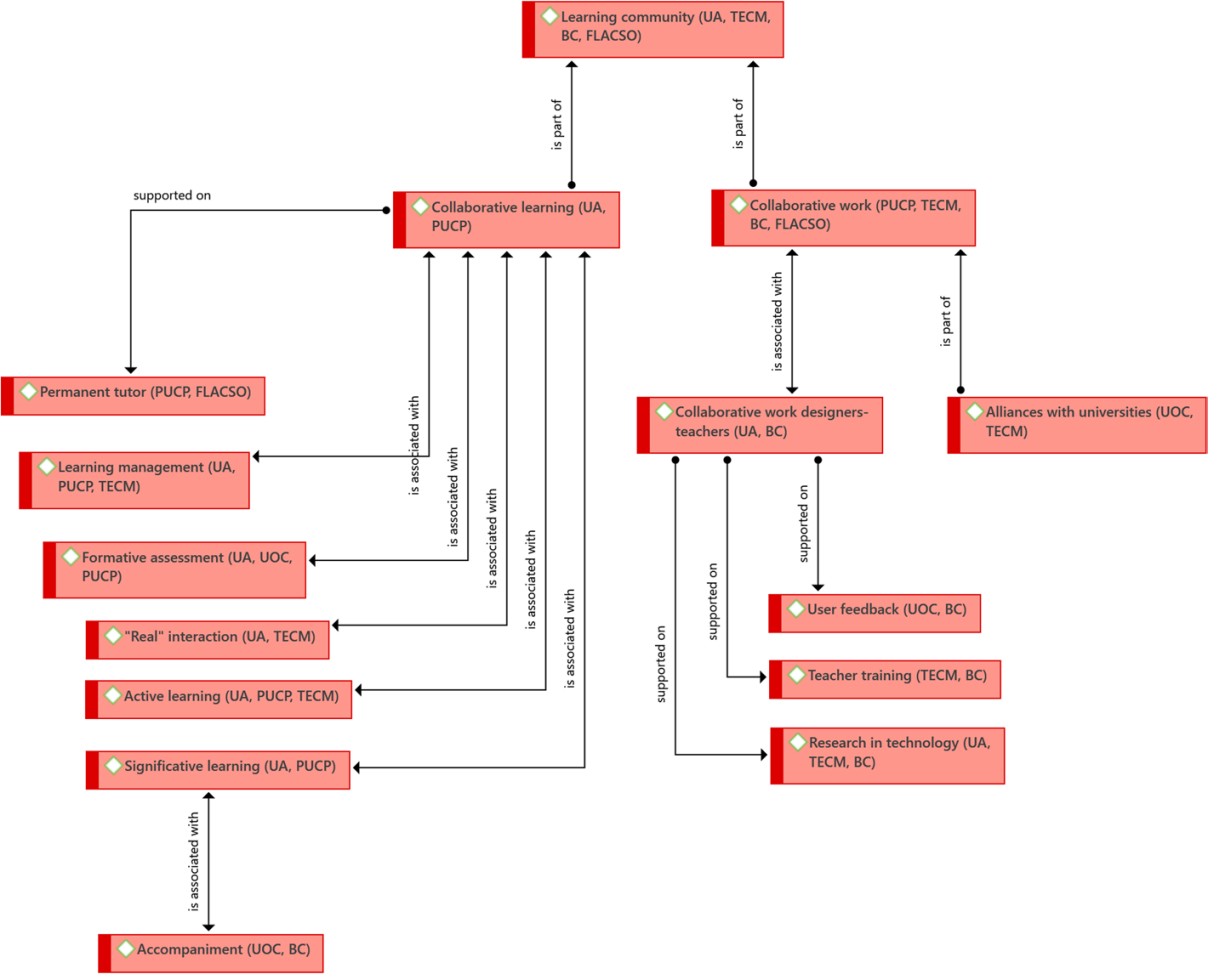
Structure of driving ideas that are shared key factors from the educational dimension. Source: Diógenes Carvajal

The relationships between the educational success factors shared by the six institutions are shown in the Fig. 4 network:

A shared key success factor is not one that is common to all institutions but one whose presence allows the eLearning or bLearning program to achieve the expected achievements. Qualitative analysis moved away from the representativeness of each factor (in quantitative terms, this means the greater frequency of occurrence), and focused on the role that each factor played in relation to the other factors, from a conceptual and structural point of view. Even so, the importance of each factor within each experience was recognized, so at the end of the name of the factors, an acronym was included that gives an account of the institutions in which each factor is present.

In the case of key success factors in the educational dimension, it was identified that the central core idea is the formation of a learning community. This is not because it is formally established, but rather because of the way in which the actions that different actors must carry out in different areas related to the eLearning and bLearning programs are articulated; hence, two elements that contribute to the formation of said community have been identified, one related to the administrative-labor, and the other to the academic.

The first aspect is collaborative work––we understand an expanded conception of the term that goes beyond pedagogical strategies that enable student learning (although it also includes it), and enters into the collaborative aspect within the institutional field. Thus, collaborative work accounts for the way in which various institutional actors relate to each other to achieve the objectives of the eLearning and bLearning programs.

One part of the collaborative work refers to the designer–teacher relationship. These two actors must work hand in hand, as teachers have knowledge about the learning expected from their students and the strategies that make such learning possible, and designers know the tools and ways in which pedagogy and technology could support the achievement of said learning. It is also necessary to know about the population to which each program is directed, not in general terms but in terms of cohorts, since each one may have particularities that require adjustments in the tools. This alliance, to give it a name, is supported by three key elements: user feedback, teacher training, and research in technology. Permanent user feedback enables problem solving and the creation of solutions; teacher training, essential for them to develop skills in the technological tools they will use, and research in technology, which will allow the designed program to be at the forefront in terms of useful and necessary tools for an eLearning or bLearning program.

The other element of the learning community refers to learning itself, and we call it Collaborative Learning. The bet on successful educational programs is inclined towards the involvement of participants in activities conceived by collaborative learning–pedagogical strategies that go beyond the presentation of resources or the completion of individual online activities, and focus on, what we could call, online learning experiences. Activities that involve joint work between the participants and the teacher and/or tutor, result in the achievement of the proposed learning objectives.

An important aspect is the presence of a permanent tutor. This person accompanies the students throughout their training process. He/she knows the students' process, their progress, and needs, and interacts with the teacher and even the designers of the programs, to solve these needs. A key aspect of the tutor is that he/she contributes to maintaining the course program, so that the participants understand how it is designed and the way in which the various subjects that comprise it are articulated. As can be inferred, it is ideal that the tutor is not a teacher of the program (although they may be). They should be someone who participates in its design, knows its structure, course, the expected learning; and who has the ability to generate empathy with participants and teachers.

This allows what we call “real” interaction, in the sense that there is permanent communication and contact between participants, tutor, and/or teacher. One of the many challenges of the eLearning and bLearning programs is the distance that is generated by not having face-to-face interactions, which creates the feeling of “abandonment” on the part of the participants. Teachers must guarantee this permanent accompaniment during the implementation of a subject, and by a tutor throughout the entire program.

Learning management is another key element. It corresponds to a coherent and clear educational program design in terms of the chosen learning model and the way it should be implemented. Additionally, formative assessment, which, in line with the “real” interaction, allows the participant to know how they are progressing in their learning process and, at the same time, allows teachers and designers to make the necessary adjustments when general needs are identified. Lastly, active learning, in the sense that participants should be challenged with pedagogical proposals that involve them beyond completing online exams, for example.

### Findings concerning the technological perspective:

Technology is a fundamental element in digital transformation and goes far beyond endowment and access. As mentioned before, technology integration must be aligned with educational strategy and its proper use requires it to be embedded within the institutional culture. We answer the following questions to gain a better understanding: What do they do in technology integration? How do they do it? What are they based on in technological strategy? What are the key success factors in this dimension?

#### What do they do in technology integration?

**Fig. 5:**
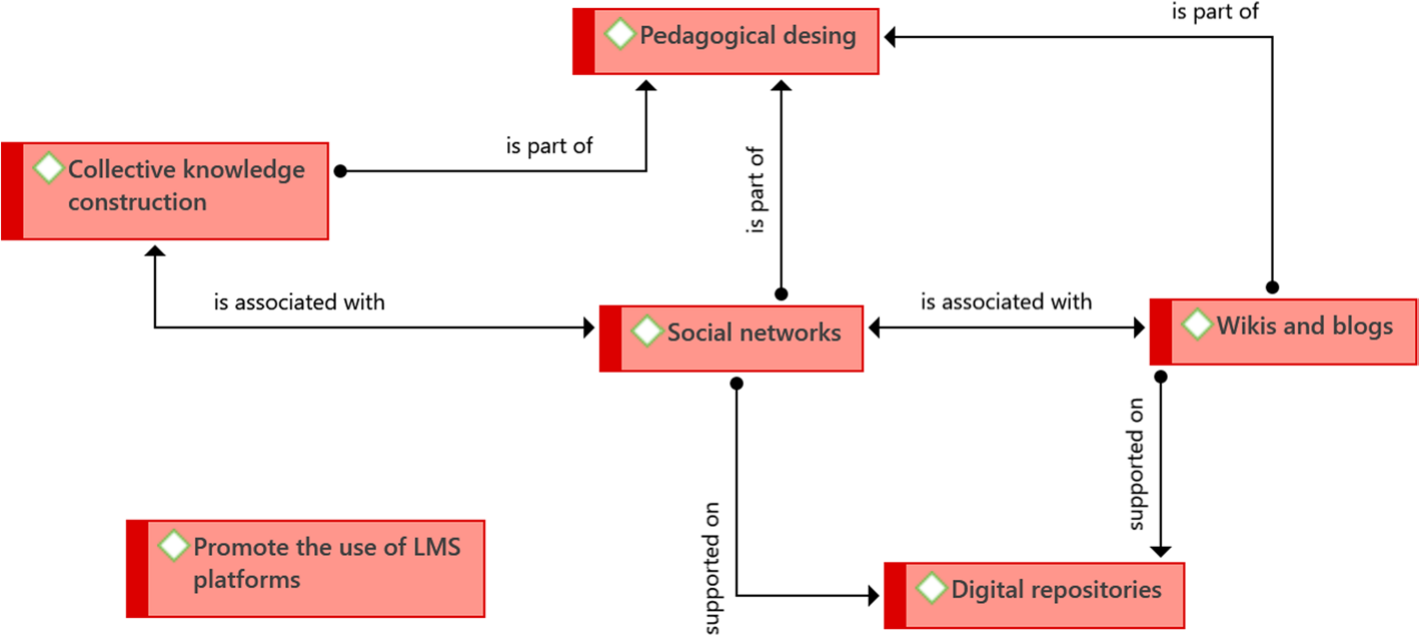
Structure of driving ideas associated with what they do in technology. Source: Diógenes Carvajal

Regarding the use of technology, six main ideas were identified. The first are social networks, which are mainly those that exist outside universities (Facebook, mainly), or that are created by the same institutions. The usefulness of social networks as support for the educational process in the eLearning and bLearning programs lies in that they allow students to be summoned to spaces for participation in which ideas are shared and discussed. Eventually agreements are reached from collaboration and discussion. The interaction spaces that these networks provide enable the collective construction of knowledge—one of the key elements identified in the educational findings (Fig. 5).

Although social networks are considered as spaces for interaction without being distinct from the training program, particularly at the UOC, we propose that they are particularly useful in programs such as communication. Sometimes, social networks, more than just being spaces for interaction, fulfill the function of being repositories of support material for the programs, which is the case of Youtube.

One element that is closely linked to social networks, and that constitutes the second finding, is the use of wikis and blogs. Although we propose that social networks allow interaction between students with a view to the collective construction of knowledge, this constructed knowledge is materialized, also collectively, with tools such as wikis and blogs, more in the former than in the latter. Blogs initially allow students to present their ideas and approaches individually, and configure spaces in which they can get feedback from their classmates and teachers—the process consists of giving an opinion and then receiving feedback. Wikis, unlike blogs, are spaces for collaborative construction, and various students take part in their development. They involve a process of collective construction of knowledge that is more direct than what is involved in blogs. However, the key for both tools is the possibility of interacting with others and generating spaces for discussion that allow progress in students’ educational process so they achieve their learning objectives. It should be remembered that the design of the program is what guarantees the achievement of these objectives.

Social networks, wikis and blogs provide collaboration spaces required for the development of the activities proposed in the programs; likewise, they enable the formation of learning communities, in which students advance as a team to achieve the learning objectives and carry out collaborative work to support their process. Additionally, student integration is also achieved; all of the above are key elements identified in the educational findings, which are possible given the existing technological support.

It should be noted that once again the element of collective knowledge construction arises, which had already been presented in the section corresponding to educational findings, as well as the articulating axis of social networks, wikis, and blogs.

As previously stated, social networks also serve as repositories of support material for programs; however, there are also digital repositories that are resources to which students have access, mainly academic publications of several kinds, which are not specifically designed for the programs but are part of the resources available to all university students. The fact that these resources are digital guarantees students on the eLearning and bLearning programs have access. Within the digital repositories, interactive games that support self-learning, videoconferences, and explanatory videos, etc. may also be included, regardless of whether they have been designed for the programs or are external. In the case of Tecnológico de Monterrey, students have access to digital laboratories that allow them to recreate their theoretical knowledge in virtual environments—there are virtual laboratories for languages, physics, chemistry, robotics, and automation.

Given that all the elements mentioned must be organized around the same space (even if they are external resources to the programs), universities have made an effort to promote the use of LMS platforms. The promotion focuses on presenting these platforms as tools that support the teaching and learning process through the resources they contain, as well as visualizing them as a competitive advantage in professional training for students.

In the specific case of UNIANDES, there is simultaneous use of two LMS platforms for formal programs: Blackboard and Moodle, which students can access from their computers, tablets, or mobile phones. These platforms contain forums, wikis, blogs, homework, exams, and grades as they are linked to the university's registration system. Uniandes promotes the use of platforms in all programs, both face-to-face and eLearning or bLearning.

In the previous section, it was identified that the design of the program is key to achieving the learning objectives. As part of the findings in the technological section, this is expanded to the pedagogical design, since we consider that the technological and the pedagogical strategies must be closely linked in the programs and courses—both eLearning and bLearning. Although the pedagogical design is a key element in all the cases studied, in UNIANDES there is a detailed description of what it implies: the pedagogical design defines the digital resources that it is convenient to use to achieve the learning objectives, including the activities that are carried out in person and virtually (in the case of bLearning programs). This is also true for learning strategies, regardless of whether they focus on active or conventional pedagogies. The profile and teaching role are defined in the pedagogical design, as are the activities that enable meaningful learning.

#### How do they do technology integration?

**Fig. 6:**
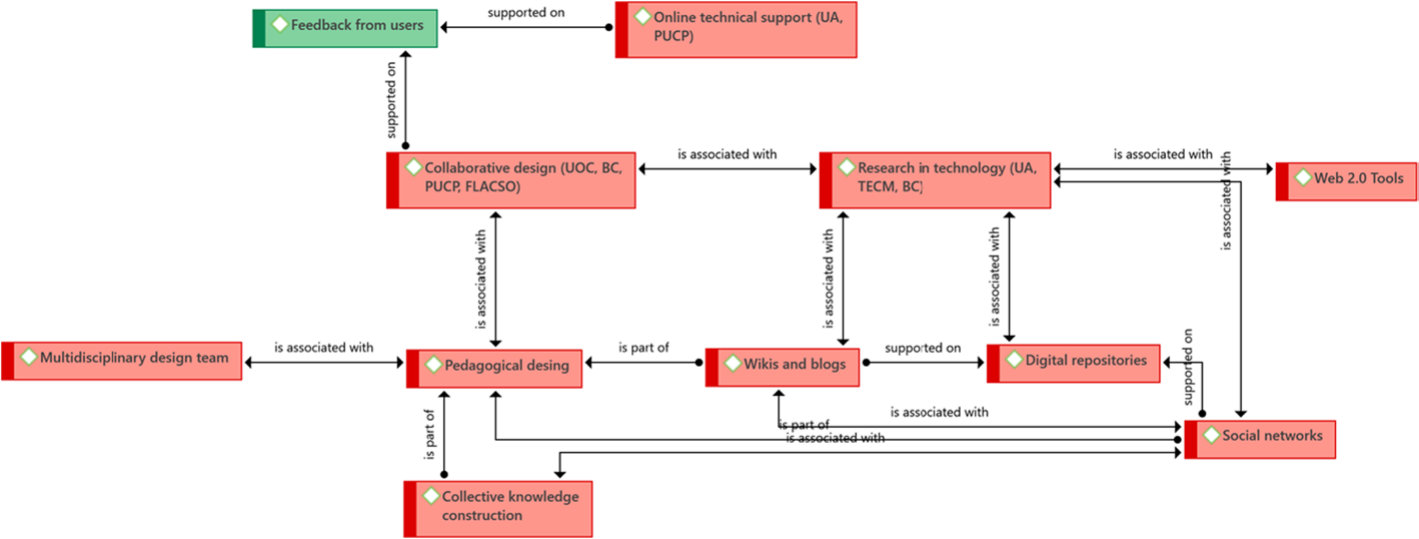
Structure of driving ideas associated with how they do technology integration. Source: Diógenes Carvajal

The main element found in this section is one that also emerged in the educational findings: collaborative design. As this element has already been addressed, we only need to state that the joint work of teachers with instructional designers is key—in terms of identifying pertinent computer tools and adequate pedagogical strategies to support the teaching and learning process. Special emphasis is placed, in this case, on the constitution of a multidisciplinary design team. Although, in each university, the teams have different compositions, it can be identified that, for the success of the programs, there must be at least two large teams: one focused on technology and one focused on pedagogical-disciplinary matters. The first team is formed by instructional designers, graphic designers, web and multimedia programmers, as well as experts in the use of technology in education, and researchers of these technologies. The second team, focused on the pedagogical issues, is formed by teachers who are knowledgeable about their disciplines and specific fields, and experts in pedagogy. The instructional designer is the bridge between both work teams (Fig. 6).

The experiences of the universities show that, beyond having programs designed taking into account all the factors associated with their success, another key element is online technical support. It is not enough to provide training in the use of the tools at the beginning of the programs—there must be online technical support throughout, since students may require support at any time regarding the use of computer tools. Similarly, in the design phase of the program it is necessary to test the tools with groups of students. This seeks to be sure that tools not only fulfill their educational purpose, but that it is also easy for students to learn to use them; the deficiency in the use of a tool should not become an obstacle for an apprentice to advance in their learning process within the program. In other words, it is necessary to take into account that the support tools that are included involve additional learning, which the students are expected to achieve in the program—hence the importance of permanent online technical support. In the case of Uniandes, this support is provided 24/7 for the two LMS platforms used by the university.

Particularly, in the latter case, permanent use is made of Web 2.0 tools, according to the functional requirements that arise in the pedagogical design. These tools allow interaction between all the participants of a program, which links this element with that of social networks, wikis, and blogs, among others. However, these tools are tested and evaluated before they are implemented (research in technology), subjected to pilot tests by teacher and student users, and permanent support is provided to solve use problems.

Other elements found, and that are already present in previous findings, are research in technology, the use of wikis and blogs, central support in social networks, and the promotion of LMS platforms by universities.

#### Key technological success factors

**Fig. 7:**
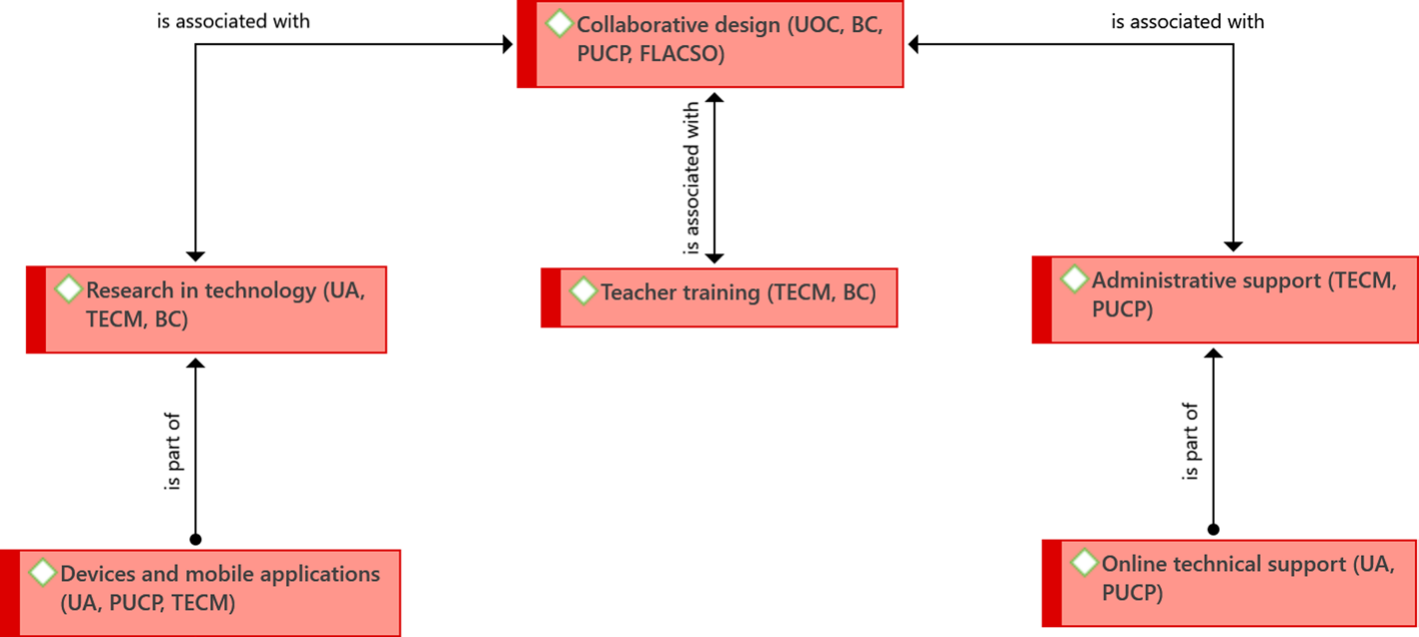
Structure of driving ideas associated with shared key success factors in technology. Source: Diógenes Carvajal

In technology, similarly to in education, key successes and individual factors were found (Fig. 7).

We identify six key success factors in technology common to the institutions. The one that we consider central is collaborative design, which is not only evident in almost all of them, but it has previously been evidenced as a key constitutive element in all areas. Including teachers, instructional designers, graphic designers and programmers, as well as taking into account the feedback that comes from the students and the teachers themselves, allows the eLearning and bLearning programs, in the technological field, to advance as expected. Of course, this factor is associated with others of vital importance.

Research in technology is essential. Not only so there is access to an adequate hardware and software infrastructure for the programs, but also to computer tools and LMS that allow the learning objectives of the programs to be achieved. Centers or units dedicated to this type of research play a fundamental role, combining two dimensions: technology and pedagogy, it is to be expected that the incorporation of technology responds to a pedagogical need, in terms of meeting the learning objectives, but also to meet the needs of access to the programs.

Due to the above, it is visible in the analysis that technology research includes devices and mobile applications, to the extent that they will allow students to access the programs without the limitations of necessarily having to do so from a desktop or laptop.

Another important element of collaborative design is teacher training. In addition to training teachers in aspects such as the design of their courses for virtual modalities, it is necessary to train them in the use of technological tools that best suit the needs of their courses. In this way, they themselves will be able to make decisions about which of these tools really allow students to learn, or even participate in the design of new ones that meet that objective.

No less important is the administrative support, which guarantees that the technological area, together with teachers and other actors involved in the programs, can advance as expected. Here it is essential, in relation to teachers and students, to have technical support online, preferably 24 h a day, since it is necessary that technical problems that may arise can be solved promptly, particularly when participants reside in areas with different time zones, such as at the UOC and at Babson College.

### Findings in the organizational dimension

The organizational component makes the digital transformation strategy viable by taking into account the human and structural elements that articulate the different roles. As noted before, organization must be aligned with education and technology. For a better understanding, these questions are answered: What do these cases do organizationally? How do they implement the organizational dimension? What is the organizational shift based on? What are the key success factors in this dimension?

#### What do these cases do organizationally?

**Fig. 8:**
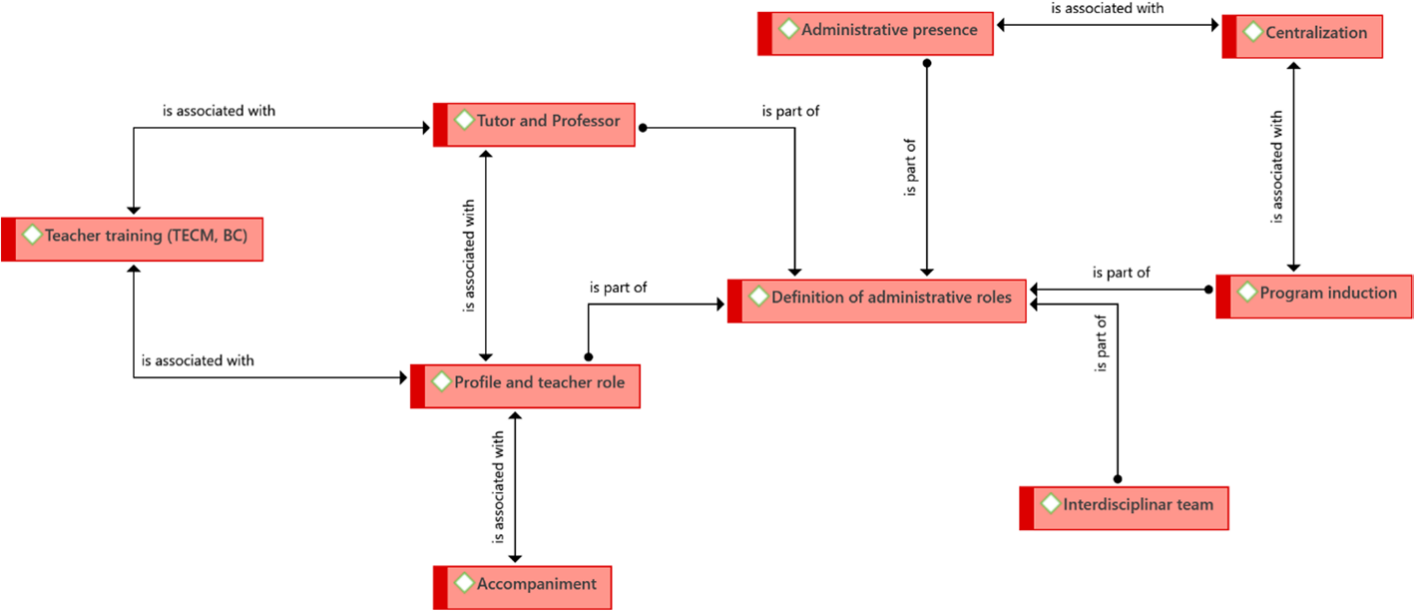
Structure of driving ideas associated with the organizational perspective. Source: Diógenes Carvajal

The definition of administrative roles is a central point from the organizational perspective; this element is common (with different names) in all the educational experiences reviewed. It is evident the need to define administrative roles and shared responsibilities for eLearning and bLearning programs. This definition is closely related to the following:

It is necessary to define the profile and teacher role, which had already been identified in the educational analysis; it is necessary to be clear about the teacher's profile, academically, but also about the role that they should play in supporting the design of the program and during its implementation. In the latter case, the FLACSO experience has led them to form a collegiate structure of tutors with regular weekly meetings to monitor students (Fig. 8).

The role of professor emerges differentiated from that of tutor; this is specific to the Tecnológico de Monterrey, where the professor (with a doctorate) is responsible for a course and coordinating the tutors, while the tutor (with a master's degree) monitors and accompanies the students, and is also responsible for adjustments to the course design. Both are in permanent contact with the students. Additionally, the latter also has access to a counselor throughout the program, who supports them regarding academic or personal difficulties that affect their performance.

The centralization of the design of the programs in a single unit has been successful. Although this leads the process, it does not work in isolation from other university units, particularly the academic units that offer training programs. The design and implementation of eLearning and bLearning programs cannot be considered in isolation by a single university agency; in fact, part of their success lies in the formation of interdisciplinary teams, in which specialized teachers and advisers participate (in instructional design and use of educational technology, among others).

The design of a Program induction also seems to be important so that students know the tools and dynamics that they will have to use and assume during their studies. In the case of PUCP, the induction lasts up to one week, and is assumed by the tutors directly. This induction program is supported, later, in the permanent accompaniment of the students, which has already been mentioned in the educational findings.

Last, but not least, an additional element is teacher training. This seeks to prepare teachers and tutors in the key tools to carry out their work. The costs of these courses are assumed by the agencies and are mandatory for teachers who join the virtual programs.

Some other key elements particular to institutions, and worth mentioning, are:

- To guarantee access to information by students; hence, it is necessary for information systems (such as libraries) to be online and their access to be allowed to all students enrolled in the programs.
- To guarantee the recovery of the investment made in the design of the courses; the designed courses must have a minimum number of students who take them, in order to recover the investment in resources made by the universities.
- Copyright, both of the resources used in the courses and of the university’s own designs and developments.

#### How do they implement the organizational dimension?

**Fig. 9:**
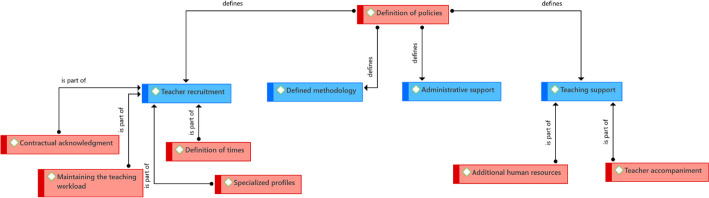
Structure of driving ideas associated with how the organizational aspect is done. Source: Diógenes Carvajal

In a similar way to what these cases do organizationally, this section refers to how they carry out the organizational aspect. It was found that the definition of policies is relevant, particularly in relation to the teaching load and the types of support that teachers will have in eLearning and bLearning programs. Institutional experiences are focused on four main themes: teacher recruitment, teacher support, administrative support, and institutional methodology (Fig. 9).

With regard to teacher recruitment, it was identified that the institutions have incorporated aspects into their recruitment policies such as respect for maintaining the teaching workload. In other words, it is intended that teachers who have one or more eLearning or bLearning courses do not have an additional burden to what they already have or to what they would have if they only had face-to-face courses. Thus, the courses in these modalities can be part of those that the permanent teachers work on; and teachers hired by the hour are paid for the planning time of the course, in addition to its implementation.

The foregoing has led to the existence of a contractual acknowledgment for teachers on the time involved in the courses beyond implementation. This comes with acknowledgment of the various activities that a teacher must carry out when they are in charge of virtually monitoring students—a visibility of the various tasks present in an eLearning or bLearning course, which often go unnoticed.

For the latter, the definition of times used by teachers for the activities of their courses is essential. Organizations must recognize that planning can be more demanding than for a face-to-face course, that they need to monitor students, and guarantee the teacher that these times will be part of their workload.

The institutions also take into account that they require specialized profiles not only for the disciplines but also to support the design of the programs and their implementation, which leads to a broad investment in human resources that will serve as support for teachers and programs.

Additionally, a series of elements related to teaching support were found to assure them the ideal conditions to carry out their work. Among these elements is the hiring of additional human resources when the programs begin to grow and the demand for courses in these modalities increases. It is necessary to be able to support the design, and there must be enough staff to provide support during the implementation, both for teachers and students.

Similarly, teacher accompaniment should be guaranteed during the design and implementation. In the case of the PUCP, experience has shown that the instructional designer who accompanies the teachers in the design of the course is the ideal person to accompany the implementation of the course. This should happen at least during the first weeks, and then cede the accompaniment to the support area and the help desk, which have a general institutional functions (they attend all courses). They want to guarantee that when starting its implementation the course has support based on the design and the guidelines that support it, rather than general support.

Although so far the guidelines have focused on teachers, it is also necessary to provide it to students in terms of administrative support. In other words, beyond access to courses and support for the activities that must be carried out, students of eLearning and bLearning programs can carry out some administrative procedures, also virtually, without having to travel to the institution; for example, inquiries, requests, and payments.

The fourth element identified is the need for a defined methodology for the design and implementation of the courses. The purpose is to guarantee the quality of the courses and programs offered, defining phases and resources, as well as the minimum criteria necessary for design and implementation, and also for evaluation. In this sense, the experience of UNIANDES seems to be the clearest, since they have defined specific processes and identified people responsible, as well as a team made up of teachers and directors of the academic unit, which is supported by specialized personnel from the area that concentrates on designing the courses.

#### Key organizational success factors

**Fig. 10:**
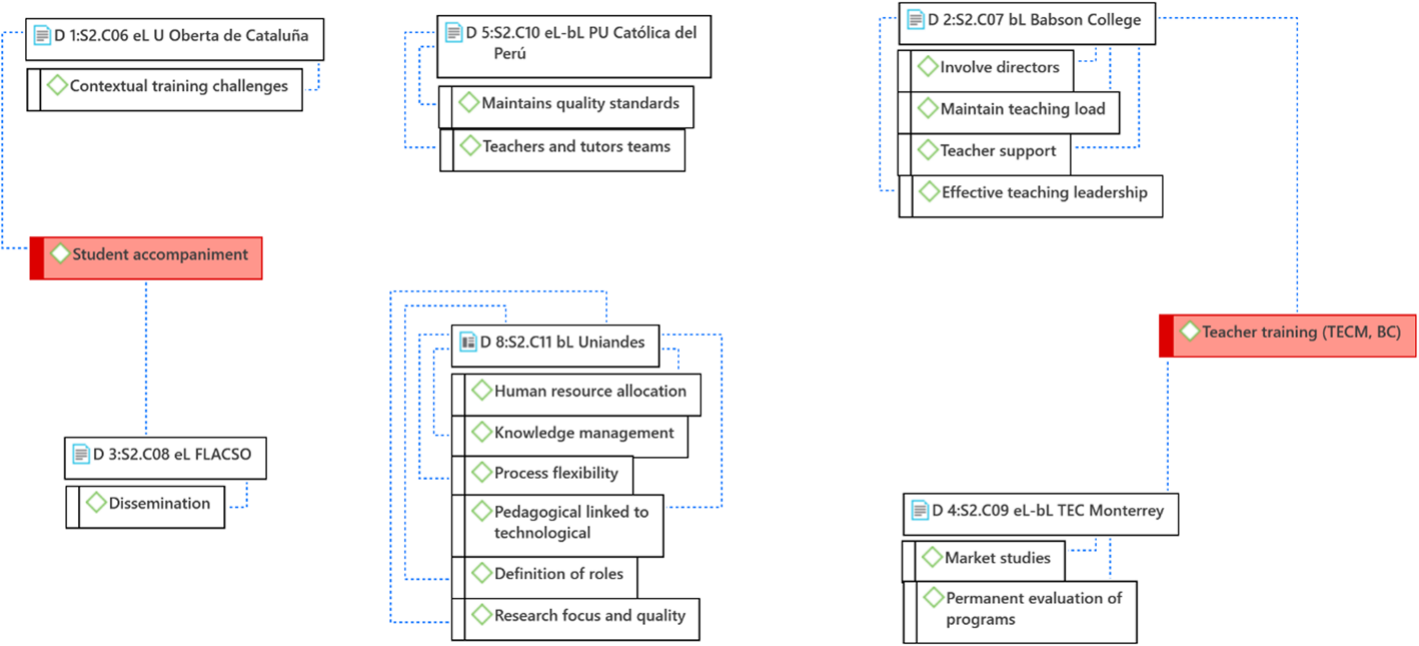
Structure of driving ideas that entail key success factors for the organization. Source: Diógenes Carvajal

When analyzing the cases, it was identified that the key organizational success factors are very diverse and particular for each organization; this is not surprising since, although higher education institutions share a similar organizational structure, their operation is different depending on the organizational culture, contextual factors, and internal policies. Hence, only two common factors have been identified between two institutions; however, this does not mean that they are not present in the others (Fig. 10).

Regarding the key success factors specific to each institution, it was found that, for the UOC, its organizational success lies in meeting the contextual training challenges—in this case, those referred to and necessary for the European context. On the other hand, for FLACSO-PENT, the key is dissemination, which can be understood as the need for teachers to participate in congresses and to publish the experience from eLearning programs. Both institutions share student accompaniment as a key factor for the success of the programs. It is guaranteed that no matter what the number of students is, (as the programs grow) they will always have support in the process. This constant student monitoring and support is something that is reinforced from the organizational point of view.

In the case of Babson College, there is a commitment to involve the directors in the use of bLearning, since both deans and vice-rectors are a fundamental support to motivate and promote participation in the programs. There is also a commitment to maintain the teaching load when they move to virtual programs and to provide teachers with the support they require to perform adequately. For this reason, effective teaching leadership has been essential, which defines courses, rules, and roles for their fulfillment, within the framework of transparency.

Tecnológico de Monterrey focuses its organizational efforts on market studies and permanent evaluation of the programs. A program is kept if it is in demand and responds to the needs of the target market. It shares, with Babson College, teacher training as a key aspect for the organizational success of its programs. There are strategies including teachers needing to be a student of any virtual program they will teach, so that they understand the proposal from the student's perspective before becoming a teacher. They can also accompany and guide them when joining the programs. All these initiatives have proven to be successful for these institutions.

In the case of the PUCP, the formation of a team of teachers and tutors who are accompanying the students and solving their concerns or difficulties in less than 24 h is fundamental. This is added to the institutional commitment to maintain the standards of the virtual programs at the same level as the face-to-face ones.

Finally, UNIANDES ensures the training of a human team that involves different professionals who work in internal teams with different but interrelated roles. They contribute with their knowledge to the design and implementation of the programs; hence, the university has designed a knowledge management system that allows all teams to keep abreast of each other's progress. These teams guarantee the permanent relationship between the technological and the pedagogical aspects. Additionally, it has the vision that evaluating the quality of the programs should be carried out from a research approach. At the institutional level, progress has been made in making administrative processes more flexible for students who enter bLearning programs, since they follow different moments and processes from those of face-to-face programs.

### The findings and digital educational transformation (ET)

The cases studied, chosen for their uniqueness in the way they manage bLearning or eLearning in higher education, serve as a framework to guide decision-making concerning the use of digital and educational technologies to promote educational transformation in similar situations. With this in mind, the tables below reorganize previous discoveries about key questions that lead the metanalysis of cases. Each array presents findings from the educational, technological and organizational perspectives, derived from previous factor analysis and its explanation (Tables 1, 2, 3).

**Table 1:** What to do for a successful institutional transformation using bLearning and / or eLearning

From the educational perspective	From the technological perspective	From the organizational perspective
E1. Define the educational model that should lead the transformation E2. Promote student collaboration by fostering learning communities E3. Cultivate meaningful learning experiences E4. Provide close student accompaniment from tutors and counselors E5. Ensure access to digital educational resources to teachers and student E6. Nurture teaching communities aligned with the educational model E7. Ensure consistency and coherence for educational program design	T1. Foster social network interaction using synchronous and asynchronous tools T2. Look after shared digital repositories of support material for programs T3. Use wikis and blogs for collective knowledge construction T4. Enrich learning experiences with expository, active and interactive tools T5. Nurture technology mediated learning communities T6. Promote the use of LMS platforms with sound educational and technological design	O1. Define profile and roles for professors, tutors, monitors, counselors O2. Centralize the design of programs O3. Promote interdisciplinary teams [specialized teachers and advisors in instructional design and use of educational technology] for program design and development O4. Include program induction and accompaniment through the process O5. Prepare teachers and tutors in key methods and tools for their roles O6. Copyright own resources and respect internal and external author’s rights

**Table 2:** How to further a successful institutional transformation using bLearning and / or eLearning

From the educational perspective	From the technological perspective	From the organizational perspective
E8. Implement collaborative strategies for learning E9. Regardless teachers may change from course to course, all of them should be inspiring and expert in their discipline E10. Accompaniment to students from tutors and counselors should be transversal, permanent and personalized E11. Student integration should nurture sense of belonging and involvement in their own learning process E12. LMS should house tools aligned with pedagogical strategies that enable learning and interactions E13. Encourage use of active learning strategies E14. Align educational design with the educational model	T7. Promote collaborative selection / design of computer tools and pedagogical strategies T8. Provide online technological support throughout T9. Test technological tools and support with representative students T10. Nurture continuous research in technologies for education and the corresponding pilot tests T11. Promote use of institutionally supported LMS platforms and interaction and learning tools	O7. State clear policies and corresponding procedures and tools for: a. Teacher recruitment, b. Teacher work load and compensation by type of contract, c. Staff support for teacher roles, d. Administrative support for online activities O8. Declare and use institutional methodology for the design and implementation of the courses

**Table 3:** Key success factors for the institutional transformation using bLearning and / or eLearning

From the educational perspective	From the technological perspective	From the organizational perspective
E16. Foster the formation of effective learning communities per course E17. Promote collaborative work among various institutional actors to achieve objectives of the eLearning / bLearning programs E18. Culture collaborative learning by using hybrid learning experiences that require “real interaction” and involve joint work between the participants and the teacher and/or tutor towards the proposed learning objectives	T12. Use collaborative technological design for desirable educational strategies T13. Conduct research in LMS, digital tools and devices, aligned with pedagogical needs, taking into consideration access opportunities and restraints T14. Provide continuous and diverse teacher training, related with education mediated with digital technologies T15. Provide 24/7 on demand online technical support to teachers and students	O9. Meet training requirements derived from applicable contextual norms O10. Ensure institutional commitment with non-face-to-face programs O11. Maintain the standards of non-face-to-face programs at the same level of the face-to-face ones O12. Perform periodic needs assessment and program evaluation to support tactic and strategic decision making O13. Provide effective student accompaniment O14. Train teams for interdisciplinary design of programs and flexible and efficient students accompaniment

These arrays include answers to table heading question, from three complementary perspectives: educational, technological, and organizational. The reader should compare these results with his/her own case:

1. WHAT TO DO for a successful institutional transformation in bLearning and / or eLearning?
2. HOW TO FURTHER a successful institutional transformation in bLearning and / or eLearning?
3. WHICH ARE KEY SUCCESS FACTORS for the institutional transformation in bLearning and / or eLearning?

## From the old “normality” to the one under construction

The findings of the meta-analysis discussed in the previous section help to understand the different perspectives that allow the design, development, implementation, monitoring and evaluation of successful bLearning and eLearning courses and programs. To conclude this paper, it is interesting to establish what happened in some of the institutions studied at the juncture of the COVID-19 pandemic, in terms of the process of change towards technology-mediated education that catapulted the transition to remote education. They also searched for a new normal, given the respective health conditions.

The institutions studied have been working for years to make ET of their processes and manage change through the use of technology in many of their activities. For this reason, the 2020 pandemic meant they had made a lot of ground in terms of remote education, but important adjustments were required so that physical, mental, and organizational health did not deteriorate due to the effects of the situation.

### The UOC in the conjuncture of the pandemic

The UOC theoretically did not need structural adjustments to carry out remote education, since its method is fully online and the university community is mentalized and instrumented to make use of digital media and a combination of self-managed and collaborative processes to undertake network learning. It is a virtual community that, by the end of 2020, included more than 200,000 students, graduates, professors, researchers, and collaborators, in 87 countries. COVID 19 accelerated the need to adapt and remain active in the labor market on the part of adult students—who comprise the majority of students at the UOC. They needed to updated to the requirements of telework by the directors, teachers, and administrators of the institution, as well as to the 24/7 coexistence with the whole family in the intimate space of interaction—at least during State enforced lockdowns. There were obvious tensions involved in dealing with a virus that has had several outbreaks and that requires health care and responsible social behavior. Additional important factors were dealing with job instability and the associated tensions that may have affected the sector in which everyone collaborates.

A UOC news item (López, 2020) says that the most important challenge for online learning is to go from emergency to quality. The following paragraphs allow us to capture the meaning of the challenge:

As Carles Sigalés, researcher and vice-rector for Teaching and Learning at the UOC, recalls, “going online is not as simple as it seems. A university that offers quality online training must be organized in a completely different way, the transfer from face-to-face to remote is not automatic. It is a system that puts students at the center of learning, with implications that go beyond the mere transfer to a screen of what a professor does in a face-to-face university. “Some people have realized that launching a model like this is not easy at all and that it is not about doing synchronous video conferencing. They have seen that very detailed design and planning is necessary, as well as the elaboration of learning resources and making decisions regarding a different way of evaluating. Additionally, developing instrumental and methodological digital competence is necessary, emphasizes Lourdes Guàrdia, researcher at Edulab and deputy director of teaching at the UOC's Studies in Psychology and Education Sciences.

In view of the above, the UOC organized a series of webinars on “Emergency non-face-to-face teaching” with the participation of faculty from all UOC studies (faculties) and published a digital book (Sangrá et al., 2020). As Teresa Guasch says in her prologue, this book collects very valuable ideas and strategies "to rethink teaching from design, resources, methodology and even evaluation, in a digital context that can no longer go back to what it was" (ibid, p.20). In this sense, Josep M. Duart comments (Duart, 2020) that the cultural change towards the digital university has been a key factor of success, since in a transition involves solving the differences that the changes of roles entail. This requires cooperation between the different actors—academic and administrative, each one in his or her role, so that the remote student can be successful. Regarding the role of teachers, Sangrá says in (López, 2020) that

teachers need to increase their digital teaching competence, as well as have more training in didactic methodologies, in managing student motivation and involvement, and in “understanding that teachers are the great designers of learning scenarios also in a digital online context ”. “And above all”, he adds, “it is necessary that we develop our capacities to know how to read the indicators that indicate changes and help students to develop their profession as apprentices. It goes without saying that, to be highly effective, this training must be carried out in the same online context in which the teaching activities will then have to be designed and managed”.

### The Tecnológico de Monterrey in the conjuncture of the pandemic

Jaime Ricardo Valenzuela (2020) says that Tecnológico de Monterrey was in its second year of the process of change for the transition to the TEC21^1^ Model when the pandemic began in March 2020. The author comments that the vision and strategy of this Model builds on the institutional efforts of the last 25 years to offer student-centered education through the use of didactic techniques that promote active pedagogy, which required large-scale teacher training and different complementary efforts to change the pedagogical culture mediated through technology. This seeks to empower the faculty. The TEC21 Model added to the above a didactic learning technique based on challenges that was organized into blocks with thematic content—in a strategic alliance with training partners (companies) that had broad interdisciplinary participation. The TEC21 Model had, since 2019, been leading the different initiatives for teacher training.

The Dean of TEC Salud provided monitoring and updates on the evolution of the global epidemic. This led to the fact that, even before the national declaration on the pandemic, the Tecnológico de Monterrey suspended its activities for 2 weeks: the first, for organization and refinement of logistics and technologies, and the second, for in-service training of teachers. In the third week, remote work was safely resumed, which has been consolidated with the Conscious Return^2^ strategy. According to Valenzuela, the institutional response to the emergency was agile and scalable, as it was applied without problems at the 26 Tecnológico de Monterrey campuses. During the process, there was monitoring every fourteen days and interaction between authorities and members of the academy.

Tecnológico de Monterrey's I3 strategy (research, innovation, and internationalization) was somewhat affected by the situation. When COVID 19 happened, research at the Tecnológico de Monterrey had already been positioned with the support of seed funds for co-financing projects, as well as institutional strengthening for groups with a strategic focus that swelled their ranks with graduate students co-financed by CONACYT (maintenance funding) and the Tecnológico de Monterrey (tuition funding). This helped to achieve abundant citations with publications in well-ranked journals, and the impact factor subsequently increased. However, the pace of research slowed down, due to the Federal Government's reaction to the pandemic—to the detriment of support for the private educational sector (Valenzuela, 2020).

**Fig. 11:**
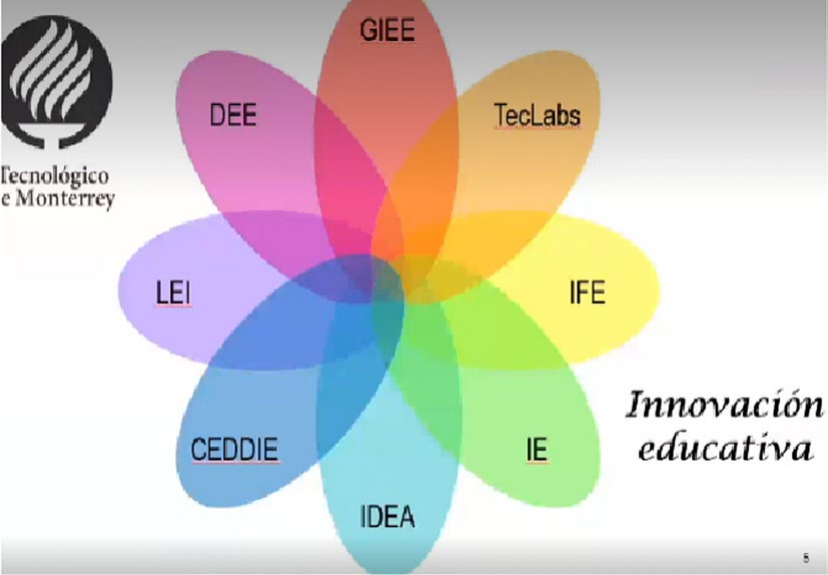
Initiatives that foster educational innovation at Tecnológico de Monterrey. Source: Valenzuela (2020)

At the beginning of the pandemic, the educational innovation strategy had a range of complementary initiatives in management and in operation, as shown in Fig. 11.

TECLABS^3^ aims to explore external cutting-edge technologies to make educational innovation. IFE^4^—Institute for the future of education, promotes educational innovation. IDEA^5^—Innovates and designs learning experiences for students. CEDDIE^6^—Center for teacher development and educational innovation supports teachers in the use of Hyflex-TEC,^7^ the hybrid and flexible model to combine digital models with different degrees of face-to-face presence.

This is complemented by the training in educational innovation offered by Tecnológico de Monterrey at the undergraduate level—LIE and doctorate level—DEE, as well as the work of the GIEE—Strategic Focus Research Group on educational innovation—part of the School of Humanities and Education.

All of the above served to support, despite the pandemic, the transition to the TEC21 Model and served as an environment for challenging work, in which teachers assume non-traditional functions by participating in its design with business partners and teach content blocks lasting five weeks. They also accompany the students in their role and in assessing challenges with the training partners. The hybrid-flexible-model of the TEC (HyFlex-TEC^8^) remodels the topology and provision of the face-to-face classrooms, with distance according to standards, in addition to guiding teachers. This model is hybrid because it combines face-to-face classes with remote ones. It is flexible since it seeks to move quickly from one scheme to another and is very TEC, since it is embedded in the LIFE experience of the Tecnológico de Monterrey.

With regard to internationalization, the pandemic led to a change in interaction between the scientific community—from mobilization and physical interaction to remote and mediated by technology. This forced us to rethink the channels and modes of interaction and enriched the process.

### UNIANDES in the conjuncture of the pandemic

The two previous experiences are a good reference to understand the Uniandina strategy to address the challenges of the pandemic. During the break week in March 2020, a multi-front process began in which we wanted to leverage each of the dimensions of an abrupt change in teaching environments, media, and strategies. We moved to remote teaching the following week, taking care of the quality of the educational process, the access to virtual environments and the physical and mental health of the members of the educational community.

From the point of view of accessibility to virtual environments, equipment and connectivity were provided to those who did not have them. The use of devices or facilities that were necessary for the correct performance of functions was authorized at home, for which the logistics team was in charge of collecting and protecting during the transfer, and delivering the equipment home.

From the perspective of occupational health, complementary and networked services were put into operation to guide processes that help to manage the tension generated by confinement, possible loneliness or family cohabitation in confined spaces, sedentary lifestyle, and loss of physical condition. This included webinars and web pages with medical, psychological, sports and cultural support services, as well as on-demand assistance for those who need it.

The academic community received support, both supply and demand, so that the transition from face-to-face to remote work was non-traumatic and increasingly effective. A good group of teachers and innovative programs had long been carrying out educational innovation mediated through the use of both technology (accompanied by ConectaTE^9^—Center for innovation in technology and education), which face-to-face courses transformed using active pedagogy. These were made virtual with relative ease, except when there was a need to obtain face-to-face access to specialized facilities (e.g., laboratories) or to be in touch with close sources of knowledge (e.g. clinical practice). Circumstances such as these could not be mediated.

The academic community also received support for the change through complementary portals that were being developed as quickly as possible. Virtualidad portal^10^ at Uniandes was the pivot of support for the transition, as it offered webinars that allowed dialogue between teachers and took advantage of pedagogical suggestions and technologies to conduct remote courses, without using transmissive models that simply use communication and information technologies to deliver and receive content. There were podcast episodes that helped to capture the meaning of pedagogical strategies supported by technology to help students in their challenges of learning in a non-face-to-face environment, either individually or in groups.

In collaboration between ConectaTE and the DOIT—Directorate for Information and Technology Services, a website was made available for teaching support^11^ with restricted access to members of the academic community, from where a portfolio of in-service training opportunities is offered. This portal also offers network support, with guidance for the best possible use of the services and digital resources set up for remote use and with articulation of initiatives of the support centers: Spanish Center,^12^ Conecta-TE, Teaching and Learning Center,^13^ Center for Applied Ethics.^14^ This initiative complemented guidance in video clips on active and alternative methodologies for teaching and learning online from the virtuality portal, as well as virtual and on-demand educational consultancies offered by ConectaTE to teachers in pedagogical, technological or digital content development. This complemented online technology trainings^15^ offered by DOIT. The above and many other efforts carried out by the Faculties and the vice-rectories, made possible the transition from face-to-face to remote teaching in courses of all levels, complementary to those already institutionalized of formal and non-formal education in hybrid and virtual modalities in which the university has been working since 2006 (Galvis, Osorio Gómez, et al., 2019).

**Fig. 12:**
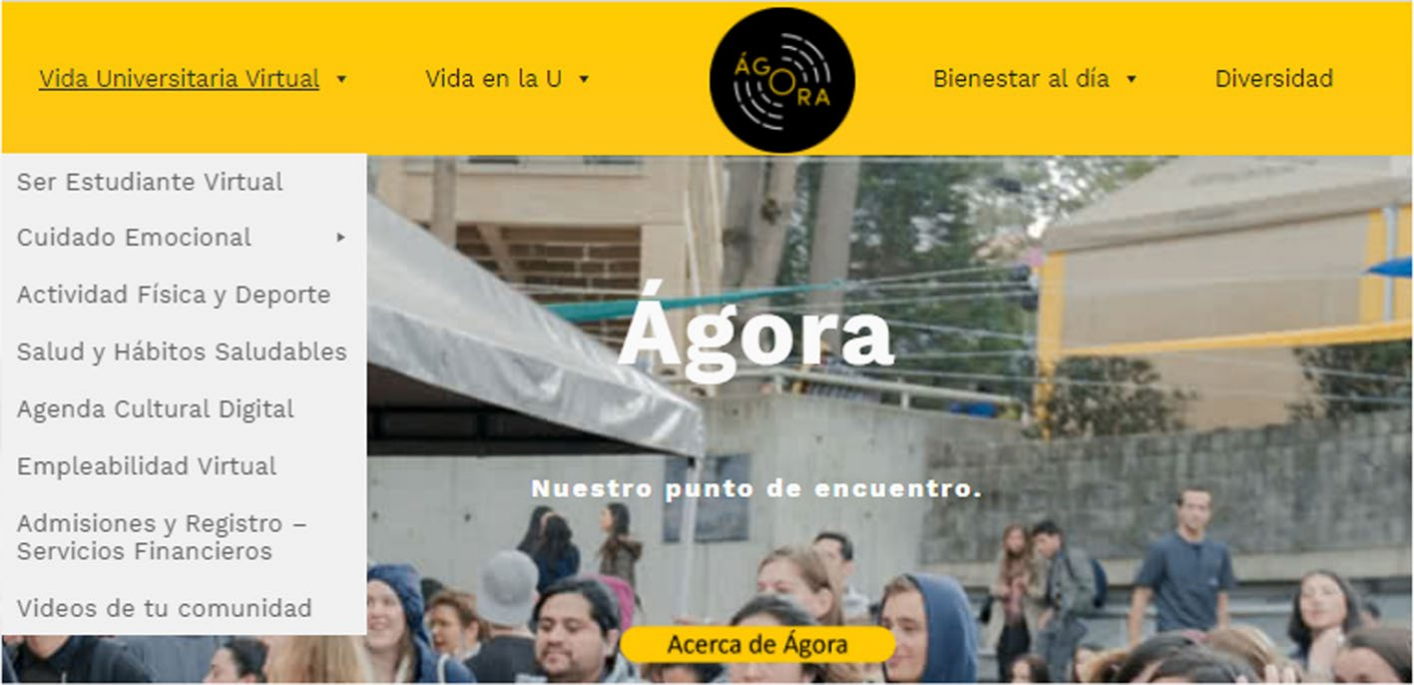
Menu for students in "virtual university life" from http://agora.uniandes.edu.co

For their part, the University students received multidimensional support equivalent to that of professors and administrators, including assurance of digital access for academic life from virtuality, plus online support for a virtual life, offered from the Agora portal,^16^ led by the DECA—Dean of Students—which is maintained in collaboration with Conecta-TE. The portal includes suggestions and resources for virtual university life such as those shown in the menu in Fig. 12, as well as suggestions of various kinds to get ahead in college, to take part in university wellness activities and services—or in campaigns of interest and promoted with the support of the students themselves through their representatives.

Raquel Bernal, the academic vice-rector, highlights the following elements through the transition process followed for the institutional adaptation to the pandemic (Bernal Salazar, 2021).

- There was a significant institutional effort to make materials, documentation and suggestions available to help learn to be a virtual student, seeking to strengthen habits to effectively assume virtual learning.
- There was an intensive task of collecting and using information through micro-surveys of teachers and students to evaluate the progress of change strategies during the pandemic, and inform the design of the subsequent stages. For example: from the micro-survey of students, the most common difficulties were taken and strategies such as the virtual student manual were designed, and specific suggestions were shared with the teachers.
- In the same way, the teacher surveys were feeding the design of the offer according to the needs and advances.
- The initiative to advance based on quantitative evidence on the student and teacher fronts is very important; this evidence was collected with high periodicity, compared to pre-pandemic monitoring practices.

Important decisions were made at UNIANDES as a result of the situation, such as the change in the learning evaluation system—which was no longer numerical; the opening of access from the network to the entire collection of digital publications from Ediciones Uniandes^17^; the open offer of webinars from the different disciplines and some continuing education courses. This complemented existing opportunities such as MOOCs^18^ offered on different platforms and participation in alliances such as TRIADA—COURSERA, which is explained below.

### Collaboration for ET in the context of the pandemic.

Various alliances have been very important to socialize and learn from other HEIs—Institutions of Higher Education, in the process of ET mediated with technology.

The alliance to share knowledge of Latin America with the world through Coursera, the Triad,^19^ is a partnership between the Tecnológico de Monterrey, the Catholic University of Chile and the University of the Andes, of Bogotá. It gives open access and free certification to members of their academic communities who take advantage of the offer of courses and specializations offered on the Coursera platform.

For its part, the University Network for Education with Technology—REDUNETE,^20^ groups together 18 public and private institutions, 17 of them Colombian and one Spanish. In alliance with the Ministry of National Education—MINEDUCACION and the Colombian Association of Universities—ASCUN, it carried out a series of complementary initiatives related to quality higher education mediated with digital technologies, which was called Educ@TED 2020.^21^ This was a virtual event with four dives of one week each and spread over two months. There was the participation of a large number of teachers and university managers in 40 virtual workshops and in six groups of plenary sessions where educational quality and different experiences were discussed, the lessons of which enrich the work in higher education; his memoirs^22^ are an important testimony. Another contribution of REDUNETE was two digital and open access books, one with recommendations to promote quality in educational practices mediated by digital technologies (Duart et al., 2020) and another with a collection of ten Colombian cases of transformative use of digital technologies in higher education (Galvis & Duart, 2020).

## Conclusions

The main aim of carrying out a comparative study of success stories in the use of eLearning and bLearning in HEIs is to support those who make decisions regarding how to be successful in the use of flexible learning environments supported by DT; it also considers how the pandemic influenced half of the HEIs that participated in the original study. Though we invite the readers to reflect on these experiences and how they may guide them in their own contexts, we want to highlight three general findings (but encourage readers to not limit themselves to them).

*The need for an educational model:* designing eLearning and bLearning programs must be done in the frame of an educational model that is defined by the HEI and is coherent in all the programs, even though they are face-to-face; the educational model embraces the ideas on how people learn and what it is necessary to do to support the learning process, the role of the many actors involved on it, and how to evaluate the learning outcomes (or competencies, which is also defined in the educational model). Active learning has proved to be effective.

*To conform learning communities:* all the actors involved in the programs must consider themselves as part of a learning community in which everyone has a role to play (teachers, instructional designers, tutors, administrative roles, graphic designers, LMS developers if needed, etc.); hence, it is necessary to define academic and organizational structures to support the programs and actors involved, guarantying that all the needs will be supplied (educational and technological resources, permanent accompaniment, among others). In these learning communities collaborative work is indispensable, and there must be permanent communication among all the actors.

*Research in technology and learning:* it is expected that HEIs are able to fund research in the use of technology to facilitate learning processes; the uncritical (or untested) incorporation of technology in the programs just to be à la mode may be counterproductive for the students’ learning process and the programs themselves. Research must be done in order to use the technological resources that best support the educational model.

Despite the findings and their usefulness, we would like to state a shortcoming of our study; as we stated in the beginning, our basic data was gathered in 2018 and the comparative study was carried out in 2019 (and some new information was gathered at the time), prior to the COVID-19 pandemic; though it was possible to gather some information on how some HEIs deal with the “new normal”, it is necessary to deepen in this aspect.

Further research must be focused on how bLearning programs adapted to the new conditions given by the pandemic (eLearning programs were not affected significantly), including the three aspects we took into account here: educational, technological and organizational conditions.

## Data Availability

Case studies object of this meta-analysis are available from Uniandes-ConectaTE.

## Footnotes

1 See http://modelotec21.tec.mx/files/folletomodelotec21.pdf.

2 See https://tec.mx/es/regreso-consciente.

3 See http://teclabs.io/es/.

4 See https://tec.mx/es/ife.

5 See https://idea.itesm.mx/.

6 See https://ceddie.tec.mx/es.

7 See http://ceddiesitio.itesm.acsitefactory.com/es/modelo-hibrido.

8 See https://tec.mx/es/hyflex-tec.

9 See http://conectate.uniandes.edu.co.

10 See https://virtualidad.uniandes.edu.co/.

11 See https://apoyodocente.uniandes.edu.co/.

12 See https://centrodeespanol.uniandes.edu.co.

13 See https://educacio.uniandes.edu.co/es/centros/centro-de-enseñanza-y-aprendizaje.

14 See https://centrodeetica.uniandes.edu.co.

15 See https://tecnologia.uniandes.edu.co/capacitaciones/.

16 See https://agora.uniandes.edu.co/vidavirtual/.

17 See https://uniandes.edu.co/es/noticias/idiomas-y-literatura/acceda-gratis-a-mas-de-500-libros-digitales-de-ediciones-uniandes.

18 See https://moocs.uniandes.edu.co/.

19 See https://www.coursera.org/programs/la-triada-egw7?authProvider=coursera4delosandes.

20 See https://redunete.net/.

21 See https://redunete.net/eventos/educated2020/.

22 See https://redunete.net/publicaciones/memorias-educated-2020/.
